# Reversing Cochlear Nucleus Maladaptive Plasticity via Customized Extracochlear Stimulation: A New Approach for Tinnitus Treatment

**DOI:** 10.1002/advs.202412349

**Published:** 2025-01-14

**Authors:** Min Chen, Shuwen Fan, Jiabao Mao, Linhan Huang, Nafisa Tursun, Chen Zhang, Wen Li, Shufeng Li

**Affiliations:** ^1^ ENT Institute and Department of Otolaryngology Eye & ENT Hospital of Fudan University Shanghai 200031 China; ^2^ NHC Key Laboratory of Hearing Medicine Research Fudan University Shanghai 200031 China

**Keywords:** cochlear implants, cochlear nucleus, gap pre‐pulse inhibition of the acoustic startle, maladaptive plasticity, tinnitus

## Abstract

Tinnitus, a widespread condition affecting numerous individuals worldwide, remains a significant challenge due to limited effective therapeutic interventions. Intriguingly, patients using cochlear implants (CIs) have reported significant relief from tinnitus symptoms, although the underlying mechanisms remain unclear and intracochlear implantation risks cochlear damage and hearing loss. This study demonstrates that targeted intracochlear electrical stimulation (ES) in guinea pigs with noise‐induced hearing loss reversed tinnitus‐related maladaptive plasticity in the cochlear nucleus (CN), characterized by reduced auditory innervation, increased somatosensory innervation, and diminished inhibitory neural networks. Additionally, a customized extracochlear ES delivered by a newly designed extracochlear electrode array to guinea pigs with salicylate‐induced tinnitus also reversed the aforementioned maladaptive plasticity and alleviated tinnitus without causing additional cochlear damage or hearing loss. These findings suggest that CI‐delivered ES may alleviate tinnitus by reversing maladaptive CN plasticity. Additionally, the extracochlear ES strategy offers a promising tinnitus treatment with minimal risk to hearing.

## Graphical abstract

An innovative extracochlear electrical stimulation strategy effectively reverses maladaptive plasticity in the cochlear nucleus of guinea pigs, providing significant tinnitus relief without inducing additional cochlear damage or hearing loss.

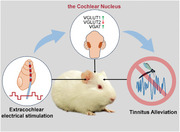

## 1 Introduction

Tinnitus, the perception of sound without external stimuli, affects millions of people worldwide and can significantly impair quality of life, often leading to anxiety, depression, and sleep disturbances.^[^ ^1^ ^]^ Despite extensive research, the precise mechanisms underlying its onset and persistence remain unclear, resulting in a lack of definitive and effective treatment options.^[^ ^2^, ^3^ ^]^ Current theories suggest that tinnitus originates from maladaptive neuroplastic changes in the central auditory pathway and other associated brain regions, triggered by reduced peripheral auditory input. This disruption leads to increased spontaneous firing rates and synchronized activity among central auditory neurons.^[^ ^4^, ^5^ ^]^ Thus, reversing these maladaptive plastic changes within the central nervous system holds promise for alleviating tinnitus.

Cochlear implants (CIs), widely used in individuals with severe to profound sensorineural hearing loss, convert sound waves into electrical signals to stimulate the auditory nerve through an intracochlear electrode array. Many patients who receive cochlear implants not only regain hearing but also experience significant relief from tinnitus, with some maintaining this benefit even after the device is turned off.^[^ ^6^, ^7^, ^8^, ^9^, ^10^, ^11^, ^12^, ^13^, ^14^ ^]^ This suggests that tinnitus relief may not solely result from the restoration of auditory input via electrical stimulation but could also involve structural or functional remodeling within the auditory pathway. However, the exact conditions and mechanisms by which cochlear electrical stimulation modulates the auditory pathway to alleviate tinnitus remain poorly understood and require further investigation. These findings highlight the potential for electrical stimulation of the peripheral auditory system, including the cochlea and auditory nerve, to influence maladaptive plasticity in the central nervous system, leading to tinnitus relief.

The cochlear nucleus (CN), the primary relay station between peripheral auditory receptors and the central nervous system, plays a critical role in processing and integrating auditory and somatosensory information. It has been implicated in tinnitus development.^[^ ^15^, ^16^, ^17^ ^]^ Type I auditory nerve fibers project to the magnocellular areas of the ventral CN (VCN) and the deep layers of dorsal CN (DCN), while somatosensory inputs from the spinal trigeminal nucleus and trigeminal ganglion primarily terminate in the granule cell domain (GCD) of the CN.^[^ ^18^ ^]^ Both auditory and somatosensory nerve fibers are glutamatergic, with vesicular glutamate transporters (VGLUT) marking their isoforms: VGLUT1 primarily labels auditory fibers, while VGLUT2 predominantly labels somatosensory fibers, though some overlap exists.^[^ ^19^, ^20^ ^]^ The maladaptive plasticity of auditory‐somatosensory innervation in different regions of the CN induced by cochlear damage has been shown to be crucial in the persistence of tinnitus in animal models.^[^ ^21^ ^]^ Maintaining the balance between excitatory and inhibitory neurotransmitters is essential for stable neural activity in the central nervous system. Alterations in inhibitory neurotransmitters, such as glycine and γ‐aminobutyric acid (GABA), within the auditory pathway have also been associated with tinnitus onset and relief.^[^ ^3^, ^22^, ^23^, ^24^, ^25^ ^]^ This underscores the importance of understanding the plasticity of auditory‐somatosensory structures and inhibitory innervation in the CN, especially as influenced by cochlear implants.

In a previous study using a noise‐induced tinnitus animal model, we demonstrated a strong correlation between extracochlear electrical stimulation‐induced changes in auditory and somatosensory innervation in the CN and tinnitus relief.^[^ ^26^ ^]^ However, these animals had nearly normal hearing, whereas most tinnitus patients exhibit some degree of hearing loss. Moreover, the causes of tinnitus extend beyond noise exposure, encompassing a wide range of factors. In the present study, we employed a noise‐induced hearing loss (NIHL) guinea pig model to investigate the effects of CI‐used intracochlear electrical stimulation on auditory, somatosensory, and inhibitory innervation in the CN. Additionally, we used a sodium salicylate‐induced tinnitus model in guinea pigs to explore the effects of a novel extracochlear electrical stimulation approach on tinnitus and its associated maladaptive changes in the CN. This dual approach allows for a comprehensive evaluation of how both intra‐ and extracochlear electrical stimulation influence neural plasticity within the CN, enhancing the clinical relevance and translational potential of our findings.

## 2 Results

### 2.1 Intracochlear Electrical Stimulation Reversed Auditory‐Somatosensory Structural Maladaptive Plasticity in CN of NIHL Guinea Pigs

**Figure 1:**
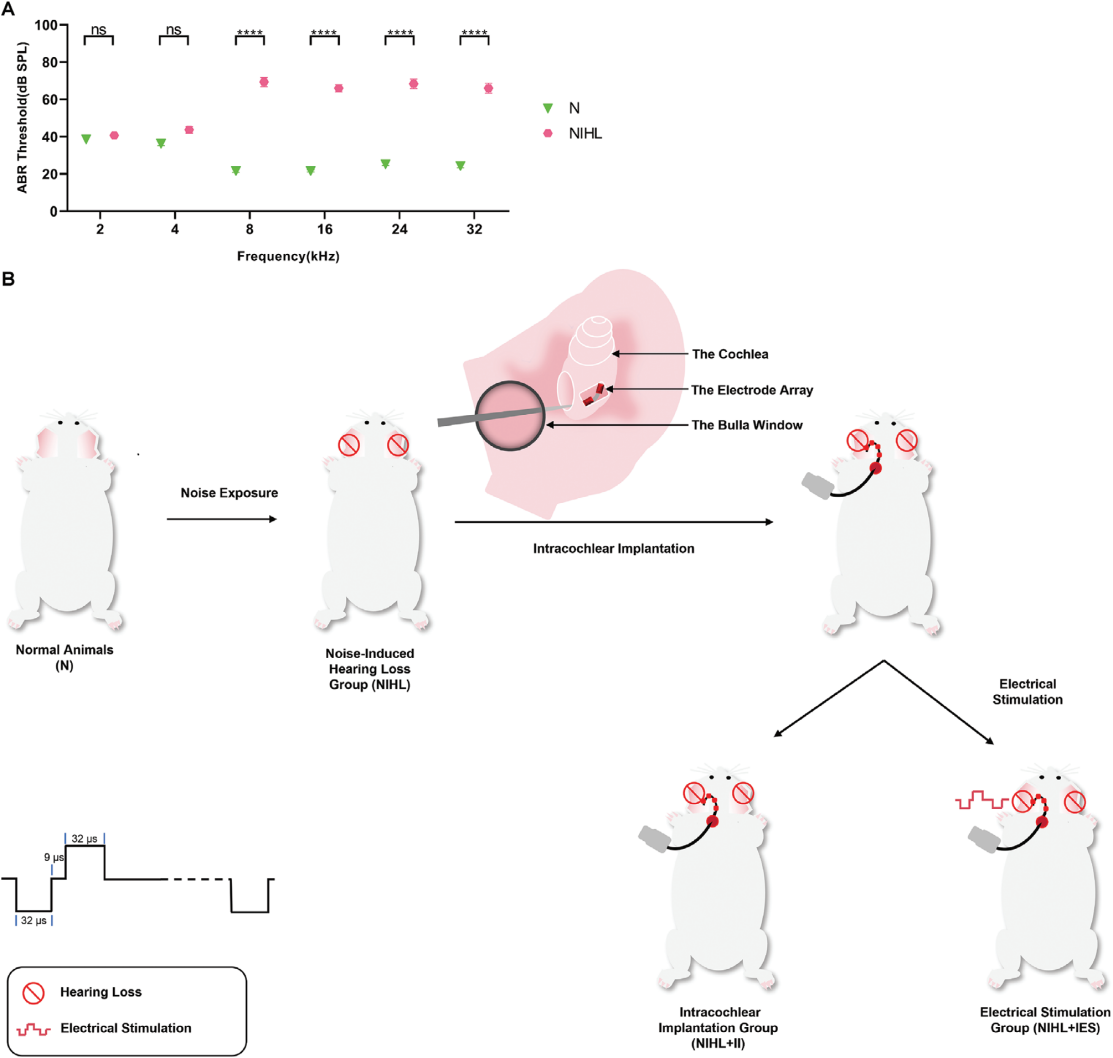
Modeling of noise‐induced hearing loss (NIHL) and experimental design of intracochlear electrical stimulation. A) Auditory Brainstem Response (ABR) thresholds of normal (N) and NIHL guinea pigs. In the comparison between the NIHL group (n = 15) and the N group (n = 15), no statistically significant alterations were observed in the ABR thresholds within the 2 and 4 kHz frequency bands, while significant elevations in the ABR thresholds were detected across the high frequency ranges, specifically at 8, 16, 24, and 32 kHz. ns, P > 0.05; ****, P < 0.0001. Two‐tailed Student's unpaired t test. Data are presented as means ± SEM. B) Experimental design and timeline of intracochlear electrical stimulation on NIHL guinea pigs. Five randomly selected NIHL guinea pigs were assigned to the control group (NIHL). Another five guinea pigs underwent unilateral intracochlear implantation of a four‐electrode array followed by sham stimulation (NIHL+II group). The remaining five guinea pigs received electrical stimulation after intracochlear implantation (NIHL+IES group). The electrical stimulation used charge‐balanced biphasic pulses with a 32‐µs phase duration and a 9‐µs interphase gap, delivered at a frequency of 12 kHz across the four electrodes.

The NIHL guinea pigs had 45–60 dB decreases in ABR thresholds at 8–32 kHz and nearly normal ABR thresholds in the 2 and 4 kHz (**Figure** **1**A). They were implanted with electrode array in the scala tympani of the cochlea to deliver intracochlear electrical stimulation. The electrical stimulation employed charge‐balanced biphasic pulses, which were commonly used in CI recipients, with intensities equal to thresholds of electrically‐evoked compound action potentials (ECAP) at a frequency of 3 kHz (**Figure** 1B).

**Figure 2:**
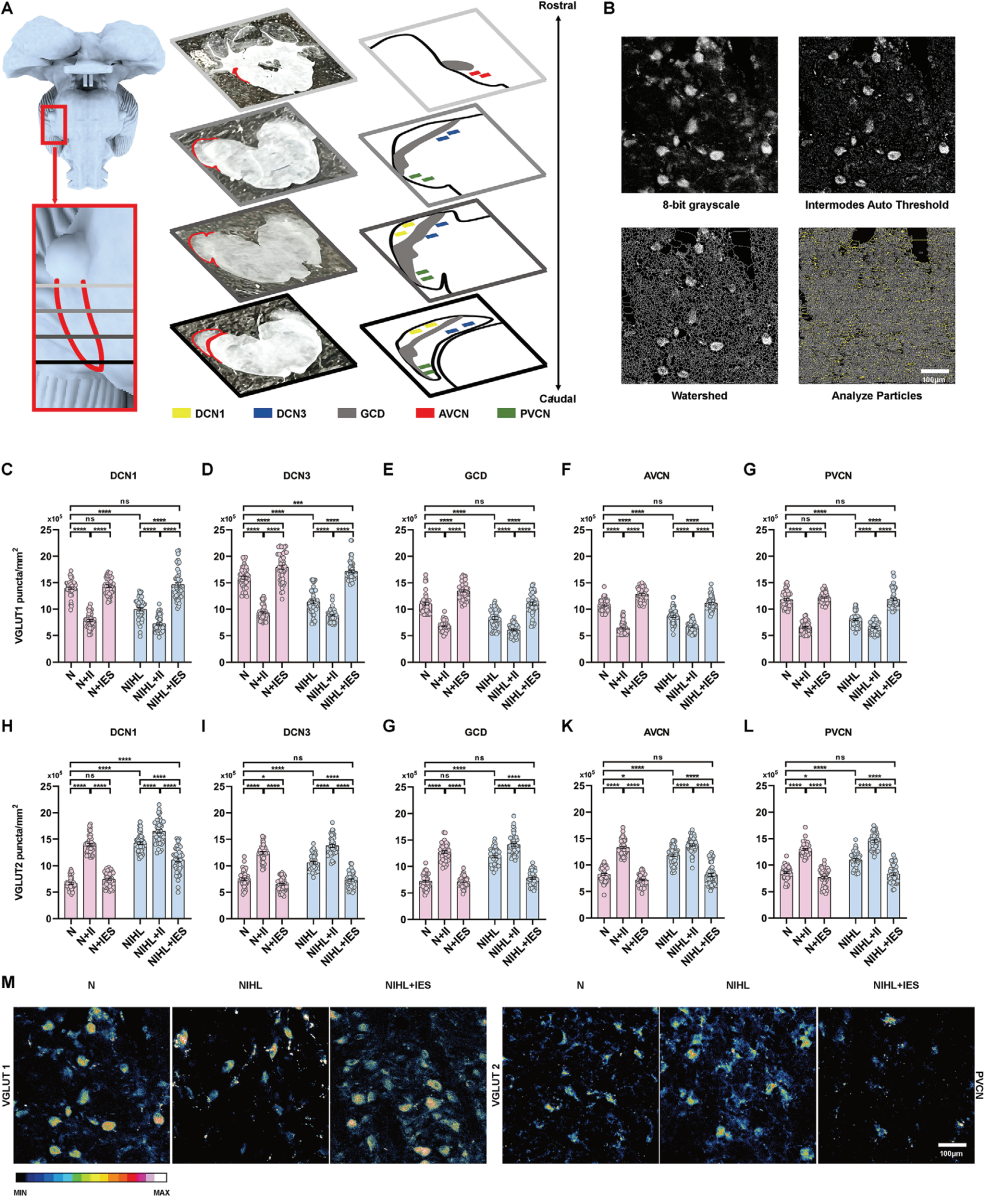
Effects of intracochlear electrical stimulation on vesicular glutamate transporter 1 (VGLUT1) and VGLUT2 density in the cochlear nucleus (CN). A) The schematic of slicing and regions of the cochlear nucleus. B) The process of calculating the puncta. The process of calculating the puncta. The original RGB images were converted to 8‐bit grayscale. Automated thresholding, specifically the “Intermodes Auto Threshold,” was applied to segment the puncta, as shown here in a 40x image. To separate fused puncta, the “Watershed” function was utilized. Finally, puncta counts were determined using the “Analyze Particles” function and normalized to the image area, resulting in puncta density measurements. C–G) Changes in VGLUT1 density in the CN. After noise exposure, NIHL guinea pigs exhibited a decrease in VGLUT1 density across all regions of the CN. In the NIHL+II group, which received sham stimulation, VGLUT1 density remained significantly reduced in all regions. However, in the NIHL+IES group, which received intracochlear electrical stimulation, the density increased above normal levels in the DCN3 region and returned to normal in all other regions. Among normal guinea pigs, VGLUT1 density decreased in all regions after intracochlear implantation (N+II group). In contrast, the N+IES group, which received intracochlear electrical stimulation, maintained normal VGLUT1 density in the DCN1 and PVCN regions, with levels higher than normal in other regions. n = 45 per group; one‐way ANOVA followed by Tukey's post hoc test. H–L) Changes in VGLUT2 density in the CN. In NIHL guinea pigs, VGLUT2 density significantly increased across all regions of the CN and continued to rise in all regions in the NIHL+II group. The NIHL+IES group showed a significant decrease in VGLUT2 density across all regions, returning to normal levels except in the DCN1 region, where it remained higher than normal. In normal guinea pigs, VGLUT2 density was significantly elevated in all regions following electrode implantation (N+II group). The N+IES group showed VGLUT2 density returning to normal levels in the DCN1 and GCD regions and even dropping below normal levels in other regions. n = 45 per group; one‐way ANOVA followed by Tukey's post hoc test. M) Representative pseudo‐colored images of the VGLUT1 and VGLUT2 immunostaining in the PVCN region of the cochlear nucleus in N group, NIHL group, and NIHL+IES group. The scale bar represents 100 µm. ns, p > 0.05; ^*^, p < 0.05; ^***^, p < 0.001; ^****^, p < 0.0001. Data are presented as means ± SEM.

The CN was divided into five distinct regions for subsequent study. These regions included the molecular layer of DCN (designated as DCN1), the deep structures of DCN (collectively labeled as DCN3), the posterior VCN (PVCN), the anterior VCN (AVCN), and the GCD. It has been suggested that the density of VGLUT1 puncta within the CN primarily reflects the density of terminals from auditory nerve fibers, while the density of VGLUT2 puncta corresponds to terminals from somatosensory nerve fibers.^[^ ^19^, ^20^ ^]^ Overall, a similar trend was shown in all the five regions. The density of VGLUT1 significantly decreased in NIHL guinea pigs, when compared to the normal hearing control group (N group). This decrement was further exacerbated in animals that underwent intracochlear implantation with an electrode array but receive sham electrical stimulation (NIHL+II group). However, subsequent to intracochlear electrical stimulation, the VGLUT1 density in NIHL+IES group significantly increased, attaining levels that were either comparable to or, in the case of DCN3, exceeded those observed in N group across all regions examined (**Figure** **2**C–G). In contrast, the density of VGLUT2 displayed a significant increase in NIHL group compared to N group. Importantly, this elevation intensified across all regions in NIHL+II group. Nonetheless, subsequent to intracochlear electrical stimulation, the VGLUT2 density in NIHL+IES group experienced a decrease in all regions, reverting to levels that were comparable to those observed in the N group in most regions. Exceptionally, in the DCN1 region, the VGLUT2 density remained elevated above normal levels, suggesting a differential response to electrical stimulation in this specific area. (Figure 2H–L). In normal hearing guinea pigs, the patterns of change in VGLUT1 and VGLUT2 densities elicited by intracochlear implantation or electrical stimulation were analogous to those observed in NIHL animals.

The results demonstrate that NIHL guinea pigs exhibited maladaptive plasticity, characterized by a reduction in auditory afferents and an increase in somatosensory afferents projecting to the CN. Intracochlear implantation of an electrode array further exacerbated these changes. Notably, subsequent intracochlear electrical stimulation effectively reversed these maladaptive alterations, highlighting its potential as a therapeutic strategy for alleviating the detrimental effects of NIHL.

### 2.2 Effect of Intracochlear Electrical Stimulation on Inhibitory Innervation in CN

**Figure 3:**
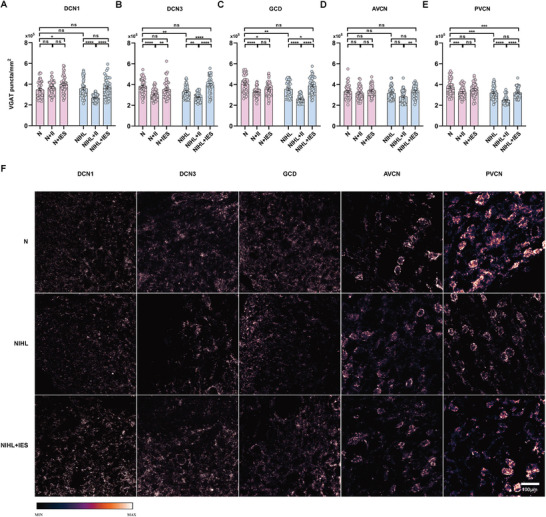
Effects of intracochlear electrical stimulation on vesicular GABA transporter (VGAT) density in the CN. A–E) VGAT densities across CN regions in various experimental groups. In the NIHL group, VGAT densities significantly decreased in the DCN3, GCD, and PVCN regions, while the DCN1 and AVCN regions remained unchanged compared to normal hearing controls (N group). In the N+II group, VGAT densities were significantly reduced in the DCN3, GCD, and PVCN compared to the N group, with no significant changes in DCN1 and AVCN. After intracochlear electrical stimulation (N+IES), VGAT densities in all regions, except DCN1 and GCD, showed no significant differences compared to the N group. In the NIHL+II group, VGAT densities significantly decreased across all regions, except the AVCN, compared to the NIHL group. After electrical stimulation (NIHL+IES), VGAT densities significantly increased in the DCN3 and GCD regions, while the other regions showed no significant changes. Notably, the PVCN region in the NIHL+IES group exhibited a significant reduction in VGAT density compared to normal levels, with no significant differences in the other regions. n = 45 per group; one‐way ANOVA followed by Tukey's post hoc test. Data are presented as means ± SEM. F) Representative pseudo‐colored images of VGAT immunostaining in different regions of the CN in N group, NIHL group, and NIHL+IES group. The scale bar represents 100 µm. ns, p > 0.05; ^*^, p < 0.05; ^**^, p < 0.01; ^***^, p < 0.001; ^****^, p < 0.0001.

We further investigated changes in inhibitory neural structures within the cochlear nucleus (CN) induced by intracochlear electrical stimulation. As shown in **Figure** **3**, VGAT densities in noise‐induced hearing loss (NIHL) guinea pigs significantly decreased in the DCN3, GCD, and PVCN regions compared to normal hearing guinea pigs, indicating a decline in inhibitory structures in these areas. In contrast, VGAT densities in the DCN1 and AVCN remained largely unchanged. After intracochlear implantation in NIHL guinea pigs, VGAT density significantly decreased across all regions except the AVCN. However, following intracochlear electrical stimulation, VGAT densities increased significantly in all regions compared to those with implantation alone. Remarkably, VGAT densities in the DCN3 and GCD of NIHL animals recovered to levels similar to those of normal‐hearing animals. While VGAT levels in the PVCN improved, they only returned to pre‐implantation levels and did not fully match those of normal‐hearing animals.

In normal‐hearing guinea pigs, intracochlear implantation led to a significant reduction in VGAT densities within the DCN3, GCD, and PVCN. The application of electrical stimulation following implantation effectively reversed this trend, with VGAT densities in the DCN3 and PVCN recovering to levels similar to the non‐implanted controls. In the GCD, although VGAT densities improved, they remained lower than in normal hearing animals. Interestingly, intracochlear implantation did not significantly alter VGAT density in the DCN1, but electrical stimulation caused a marked increase, surpassing even the levels seen in normal hearing guinea pigs.

### 2.3 Intracochlear Electrical Stimulation did not Further Impair Hearing and Cochlear Structures

The changes in VGLUT1, VGLUT2, and VGAT densities, which represent auditory‐somatosensory afferent innervation and inhibitory neural architecture, may be influenced by alterations in cochlear function and structural integrity. To evaluate the impact of intracochlear electrical stimulation on residual hearing and cochlear structure in NIHL guinea pigs, we performed ABR threshold measurements to assess changes in auditory sensitivity. Furthermore, we examined the density of paired synapses on inner hair cells (IHCs), providing critical insights into the structural integrity and functional connectivity of the cochlea.

**Figure 4:**
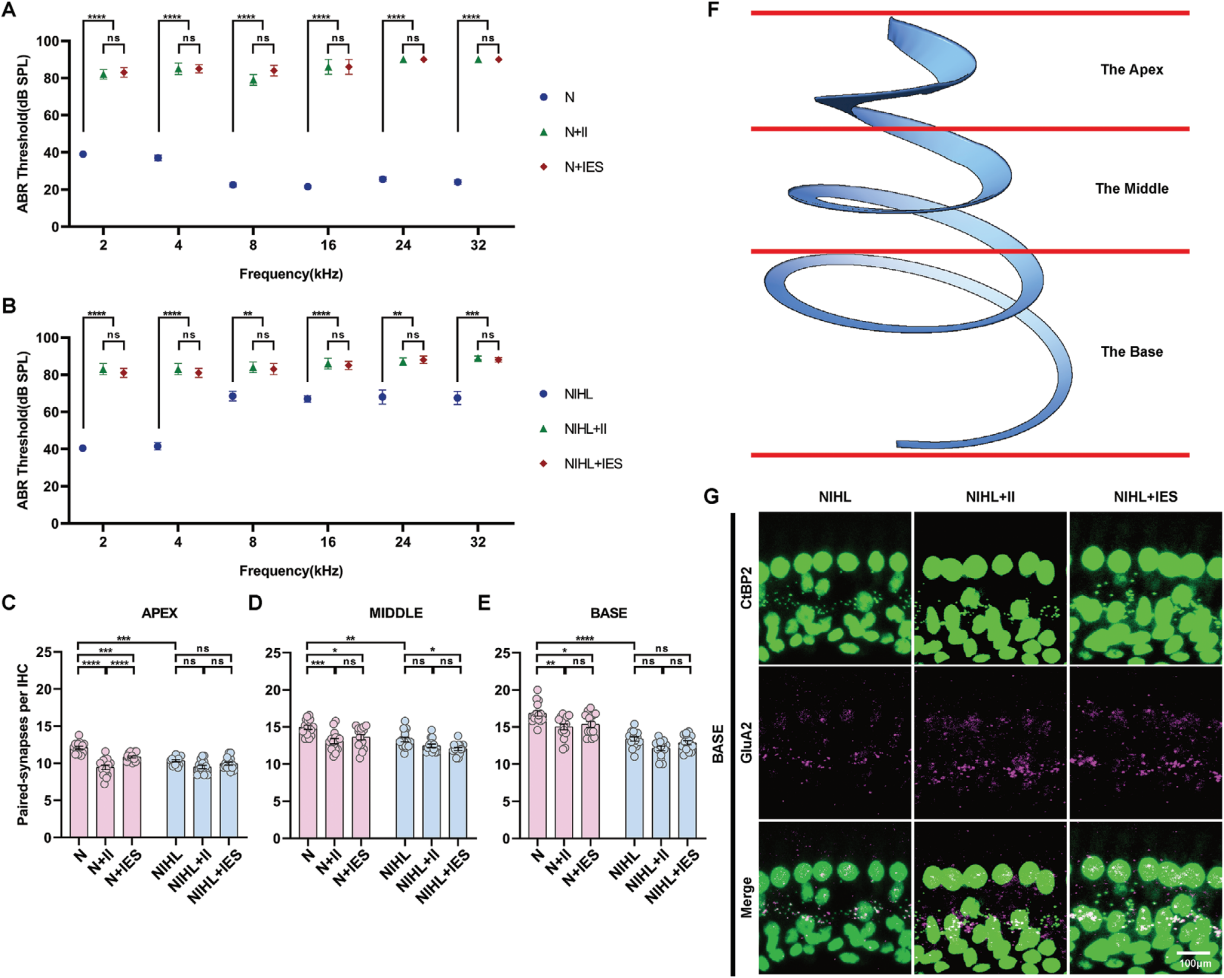
Effects of intracochlear electrical stimulation on hearing and paired synapse density in inner hair cells (IHCs) of normal and NIHL guinea pigs. A) ABR threshold shifts in normal guinea pigs. ABR thresholds in the N+II group (n = 5) were elevated across all frequencies compared to normal animals (n = 10), but no significant difference was observed between the N+II and N+IES groups (n = 5). Data are presented as means ± SEM, and a two‐tailed unpaired Student's t‐test was used for analysis. B) ABR threshold shifts in NIHL guinea pigs. ABR thresholds remained elevated at all frequencies in NIHL+II group, with no significant differences between the NIHL+II and NIHL+IES groups (n = 5 per group). C–E) Changes in paired synapse density per IHC in the apex, middle, and base of the basilar membrane. In all three regions, synapse density significantly decreased in NIHL groups compared to normal hearing animals. There was no further reduction following implantation (NIHL+II) or electrical stimulation (NIHL+IES). In normal guinea pigs, intracochlear implantation (N+II) resulted in a significant reduction in synapse density across all regions compared to the N group. Subsequent electrical stimulation (N+IES) further decreased synapse density in the apex but had no significant effect on the middle and basal regions compared to the N+II group. n = 15 per group; one‐way ANOVA followed by Tukey's post hoc test; Data are presented as means ± SEM. F) Regional division of the basilar membrane. G) Representative immunostaining images of presynaptic ribbons (CtBP2), postsynaptic patches (GluA2) and merged images to identify paired synapses. The scale bar represents 100 µm. ns, p > 0.05; ^*^, p < 0.05; ^**^, p < 0.01; ^***^, p < 0.001; ^****^, p < 0.0001.

After intracochlear implantation, a shift of ≈20 dB in ABR thresholds was observed across all frequencies in NIHL guinea pigs, while the shifts were more pronounced (8–32 kHz) in normal hearing animals (**Figure** **4**). Despite these shifts, no statistically significant differences were found between the post‐implantation ABR thresholds of the two groups across the entire frequency spectrum. Additionally, there was no significant difference in ABR thresholds between animals that received intracochlear electrical stimulation and those undergoing a sham procedure, regardless of hearing status (NIHL or normal hearing) and across all frequencies tested. These findings suggest that while surgical implantation impaired hearing, particularly in normal hearing guinea pigs with higher‐frequency sensitivity, intracochlear electrical stimulation did not exacerbate the hearing loss in either group. In essence, intracochlear electrical stimulation was not detrimental to auditory function in either cohort.

To accurately assess IHC synapse quantity, the basilar membrane was divided into three segments—apex, middle, and base—with each segment analyzed separately to capture potential variations in synapse density across frequency representations. In NIHL guinea pigs, the number of paired synapses per IHC significantly decreased in all regions compared to normal hearing animals (Figure 4A,B). In the NIHL, NIHL+II, and NIHL+IES groups, there was no significant difference in paired synapse density per IHC across regions, except in the middle region where the NIHL+IES group showed a significant decrease compared to the NIHL group. These results suggest that neither intracochlear implantation nor electrical stimulation alone led to synapse loss in any cochlear region in NIHL animals. However, their combined application specifically resulted in a loss of IHC synapses in the middle region.

In normal hearing animals, intracochlear implantation led to a significant loss of paired IHC synapses across all cochlear regions. However, in the electrical stimulation group, no further synapse loss was observed compared to the implantation‐only group (Figure 4C–E). Interestingly, at the cochlear apex, the electrical stimulation group showed a significant increase in synapse density compared to the implantation‐only group. These findings indicate that while intracochlear implantation reduced paired IHC synapse density across all regions, electrical stimulation did not exacerbate this reduction, and in certain regions, such as the apex, it may even promote synapse recovery.

### 2.4 Extracochlear Electrical Stimulation Attenuated Salicylate‐Induced Tinnitus Behaviors

Guinea pigs underwent a 14‐day regimen of continuous intraperitoneal sodium salicylate injections, a protocol designed to induce bilateral tinnitus in a subset of the animals. To identify those displaying tinnitus for subsequent experiments, the Gap Prepulse Inhibition of the Acoustic Startle (GPIAS) test was employed. To mitigate the risks associated with intracochlear implantation while preserving auditory function and cochlear integrity, a bilateral extracochlear implantation strategy was developed. This approach involved delivering extracochlear electrical stimulation at intensities based on each animal's ECAP thresholds, fixed at a frequency of 3 kHz. By adopting this method, we investigated the effects of bilateral extracochlear electrical stimulation on tinnitus behaviors while minimizing potential cochlear damage.

Twenty guinea pigs confirmed to exhibit tinnitus behaviors were randomly assigned to one of four groups: the S14 group (euthanized immediately after salicylate administration and tinnitus assessment), the R7 group (monitored for 7 days without intervention), the T+EI group (bilateral extracochlear implantation without electrical stimulation), and the T+EES group (bilateral extracochlear implantation with electrical stimulation). Additionally, five control animals received 14 days of intraperitoneal saline injections (NS group).

**Figure 5:**
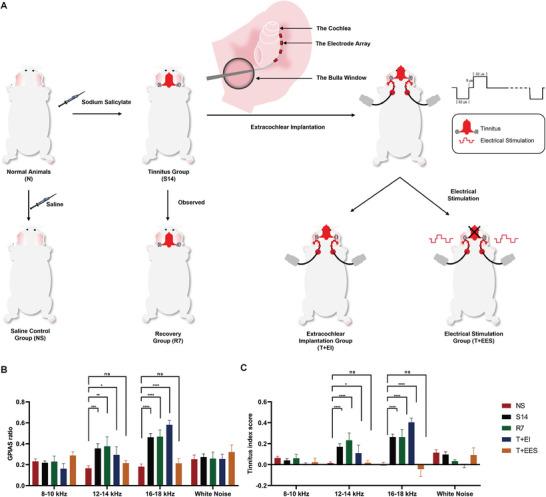
Effects of extracochlear electrical stimulation on tinnitus behaviors. A) Experimental design and timeline for extracochlear electrical stimulation in tinnitus guinea pigs. Tinnitus was induced in normal guinea pigs (N) through a 2‐week intraperitoneal administration of sodium salicylate. Five tinnitus‐confirmed guinea pigs were randomly assigned to the tinnitus control group (S14). Another five tinnitus guinea pigs were maintained without any intervention for 7 days after discontinuing the salicylate treatment (R7). Five guinea pigs underwent bilateral extracochlear implantation without electrical stimulation (T+EI). An additional five guinea pigs received electrical stimulation following extracochlear implantation (T+EES). Lastly, five animals that received 14‐day intraperitoneal saline injections served as a control group (NS). B,C) Changes in Gap Prepulse Inhibition of the Acoustic Startle (GPIAS) ratios and Tinnitus Index (TI) scores in tinnitus‐induced guinea pigs. No significant differences were observed in GPIAS ratios (B) or TI scores (C) between the groups at background noise frequencies of 8–10 kHz and white noise. However, in the S14 group (n = 5), where tinnitus was successfully induced, GPIAS ratios and TI scores were significantly elevated at background noise frequencies of 12–14 kHz and 16–18 kHz compared to the NS group. The R7 and T+EI groups showed no significant differences in GPIAS ratios or TI scores compared to the S14 group, with both remaining significantly higher than those in the NS group. In contrast, the T+EES group, which received extracochlear electrical stimulation, demonstrated a significant reduction in GPIAS ratios and TI scores compared to the S14 group, aligning with the levels observed in the NS group. n = 5 per group; two‐tailed Student's unpaired t test. Data are presented as means ± SEM. ns, *p* > 0.05; ^*^, *p* < 0.05; ^**^, *p* < 0.01; ^***^, *p* < 0.001; ^****^, *p* < 0.0001.

As shown in **Figure** **5**B,C, tinnitus animals in the S14 group exhibited significant increases in both GPIAS inhibition rates and tinnitus index (TI) scores at the 12–14 kHz and 16–18 kHz frequency ranges. However, no significant differences were observed at 8–10 kHz or in response to broadband noise compared to the NS group. The R7 group, observed for 7 days after discontinuing salicylate treatment, showed similar GPIAS inhibition rates and TI scores as the S14 group, indicating persistent tinnitus behaviors.

Following extracochlear implantation and electrical stimulation (T+EES group), a marked reduction in GPIAS inhibition rates and TI scores was observed, bringing them in line with those of the NS group. In contrast, the T+EI group, which underwent implantation without electrical stimulation, showed no significant changes in GPIAS inhibition or TI scores compared to the tinnitus‐inducing S14 group. These results suggest that extracochlear electrical stimulation significantly attenuated salicylate‐induced tinnitus behaviors in guinea pigs.

### 2.5 Extracochlear Electrical Stimulation Reversed Salicylate‐Induced Auditory‐Somatosensory Structural Maladaptive Plasticity in CN of Tinnitus Guinea Pigs

**Figure 6:**
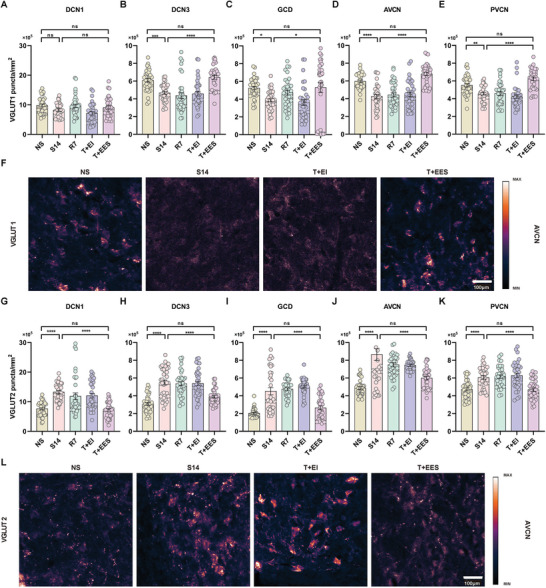
Effects of extracochlear electrical stimulation on VGLUT1/2 density in the CN. A–E) VGLUT1 density in the S14 group was significantly reduced in the DCN3, GCD, AVCN, and PVCN regions compared to the NS group. This reduction was reversed in the T+EES group, but not in the T+EI or R7 groups. In the DCN1 region, VGLUT1 densities in both the S14 and T+EES groups remained comparable to those in the NS group (n = 35 per group). F) Representative pseudo‐colored images of VGLUT1 immunostaining in the AVCN region of the CN in the NS, S14, T+EI, and T+EES groups. G–K) VGLUT2 density was significantly higher in all CN regions of the S14 group compared to the NS group. This elevation was reversed to NS group levels in the T+EES group, but not in the T+EI or R7 groups. L) Representative pseudo‐colored images of VGLUT2 immunostaining in AVCN regions of the CN in NS group, S14 group, T+EI group and T+EES group (n = 35 per group). One‐way ANOVA followed by Tukey's post hoc test. Data are presented as means ± SEM. ns, p > 0.05; ^*^, p < 0.05; ^**^, p < 0.01; ^****^, p < 0.0001. The scale bars represent 100 µm.

There were significant reductions in VGLUT1 densities across all regions of the cochlear nucleus (CN), except DCN1, in guinea pigs with tinnitus symptoms (S14 group) compared to non‐tinnitus controls (NS group). These reductions remained stable over a 7‐day period in the R7 group. Similarly, bilateral extracochlear implantation alone (T+EI group) did not significantly affect VGLUT1 densities. However, bilateral extracochlear electrical stimulation (T+EES group) following implantation led to a notable and statistically significant increase in VGLUT1 densities, restoring them to levels comparable to those in the NS group (**Figure** **6**A‐F).

In contrast, VGLUT2 densities were significantly elevated in all CN regions of tinnitus‐affected guinea pigs (S14 group) compared to the NS group. This upregulation persisted in the R7 group, as well as in the T+EI group, indicating the stability of these elevated levels. Remarkably, in tinnitus animals that received bilateral extracochlear electrical stimulation (T+EES group), the previously elevated VGLUT2 densities were significantly reduced, returning to levels observed in non‐tinnitus animals (Figure 6G‐L).

These findings suggest that salicylate‐induced tinnitus prompts maladaptive plasticity within the CN, characterized by reduced auditory innervation (VGLUT1) and increased somatosensory innervation (VGLUT2). Notably, extracochlear electrical stimulation effectively reversed these maladaptive changes, correlating with significant reductions in tinnitus‐related behaviors.

### 2.6 Extracochlear Electrical Stimulation Reversed Alterations of Inhibitory Neural Structures in CN of Tinnitus Guinea Pigs

**Figure 7:**
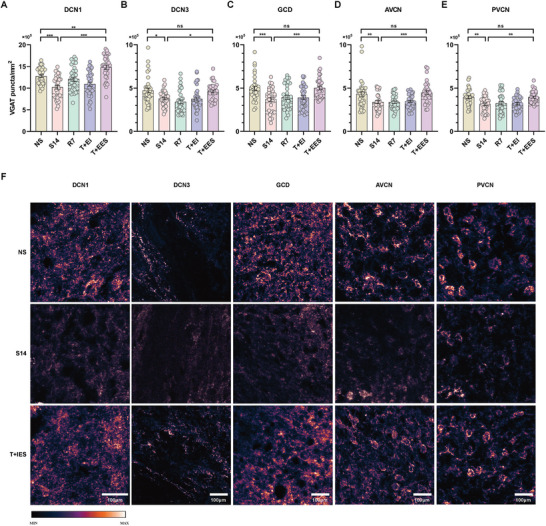
Effects of extracochlear electrical stimulation on VGAT density in the CN. A–E) VGAT density in the S14 group was significantly reduced in all regions of the CN compared to the NS group. In the T+EES group, but not in the T+EI or R7 groups, this reduction was reversed to levels comparable to the NS group in all regions except the DCN1 region, where it remained higher than in the NS group (n = 35 per group). One‐way ANOVA followed by Tukey's post hoc test. Data are presented as means ± SEM. F) Representative pseudo‐colored images of VGAT immunostaining in different regions of the cochlear nucleus. ns, p > 0.05; ^*^, p < 0.05; ^**^, p < 0.01; ^***^, p < 0.001. The scale bars represent 100 µm.

Across all regions of the cochlear nucleus (CN), guinea pigs with tinnitus symptoms (S14 group) exhibited a significant reduction in VGAT densities compared to non‐tinnitus animals (NS group). Notably, neither the seven‐day observation period without intervention (R7 group) nor extracochlear implantation without electrical stimulation (T+EI group) resulted in significant changes in VGAT densities across any CN regions. Notably, tinnitus animals that received extracochlear electrical stimulation (T+EES group) showed a significant increase in VGAT densities in all CN regions, reaching levels comparable to, and in some cases even exceeding, those of non‐tinnitus animals, particularly in the DCN1 region (**Figure** **7**).

### 2.7 The Influence of Extracochlear Electrical Stimulation on the Cochlear Function and Structure of Tinnitus Guinea Pigs

**Figure 8:**
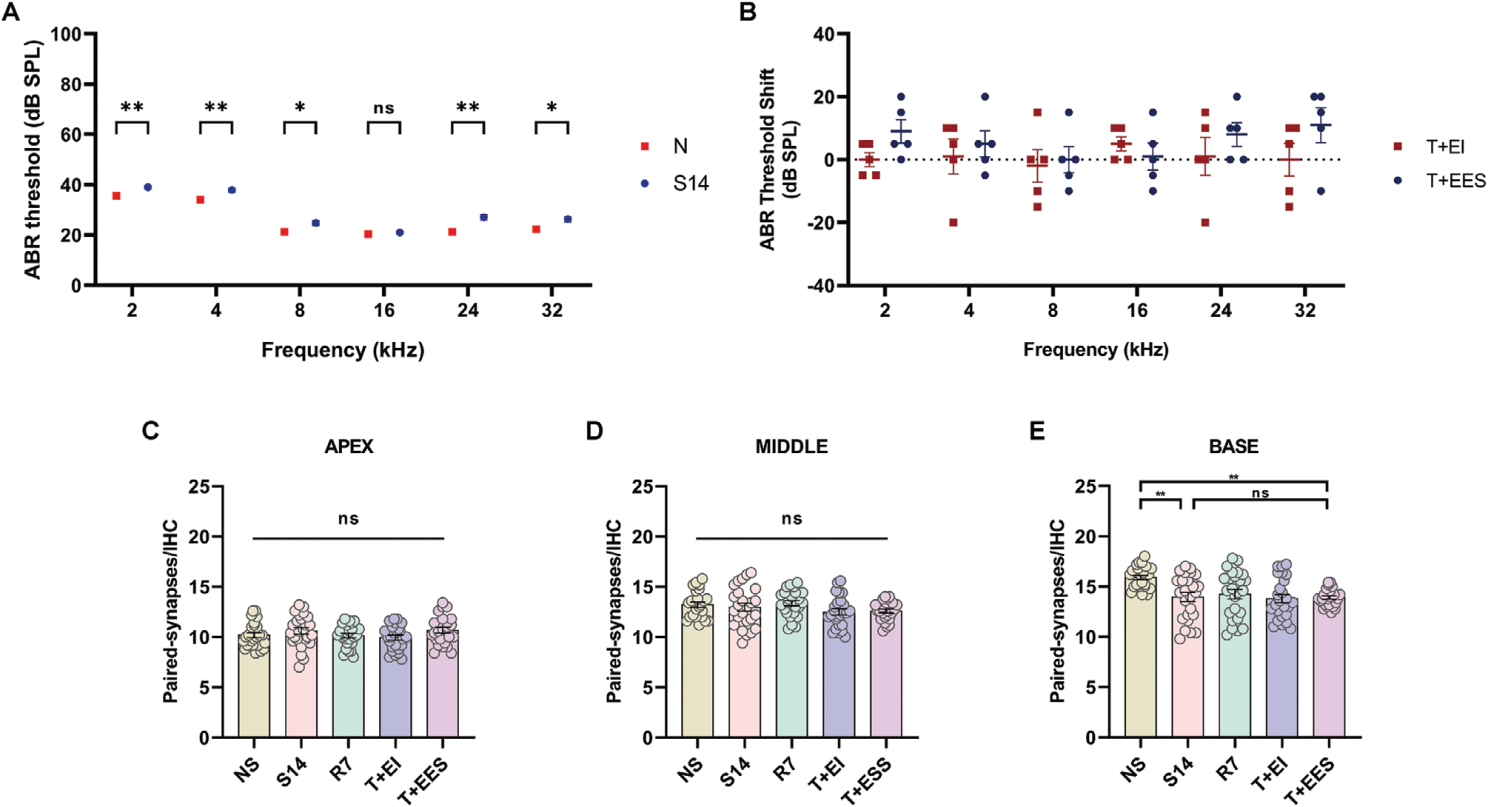
Effects of extracochlear electrical stimulation on hearing and the number of paired synapses per IHCs. A) ABR thresholds per frequency in NS group and S14 group. Salicylate‐induced tinnitus guinea pigs (S14 group) showed a slight but significant increase in ABR thresholds at 2, 4, 8, 24, and 32 kHz compared to N group. n = 5 per group; one‐way ANOVA followed by Tukey's post hoc test. B) The difference in ABR threshold shifts between the T+EES and T+EI groups compared to the S14 group at all frequencies was not statistically significant. n = 5 per group; two‐tailed Student's unpaired t test. C–E) Changes of the number of paired synapses per IHC. In the apex (C) and middle (D) regions, the synapse density in the S14, R7, T+EI, and T+EES groups did not differ from the NS group. However, in the basal region, the S14 group showed a significant decrease compared to the NS group, but with no significant difference observed between the T+EES and S14 groups. n = 25 per group; one‐way ANOVA followed by Tukey's post hoc test. ns, p > 0.05; ^*^, p < 0.05; ^**^, p < 0.01; ^****^, p < 0.0001. Data are presented as means ± SEM.

ABR thresholds and densities of paired IHC synapses were measured to evaluate changes in auditory function and cochlear microstructures in response to tinnitus induction, extracochlear implantation, and electrical stimulation. As shown in **Figure** **8**A,B, a 14‐day regimen of sodium salicylate administration induced a modest but statistically significant elevation in ABR thresholds across most measured frequencies, except at 16 kHz. Following extracochlear implantation, comparisons of pre‐ and post‐implantation ABR thresholds revealed no significant changes at 2 and 4 kHz, with slight increases at 8 and 16 kHz, and more pronounced elevations at 24 and 32 kHz. Importantly, there were no statistically significant differences in ABR thresholds between T+EI group and T+EES group across all frequencies, indicating that electrical stimulation did not exacerbate hearing loss.

In the S14 group, a significant reduction in paired IHC synapses was observed at the basal region of the cochlea, while no significant changes were noted at the apex or middle regions compared to NS group. In both the T+EI and T+EES groups, the number of paired IHC synapses in the apical, middle, and basal regions did not significantly differ from those in the S14 group. These findings suggest that neither extracochlear implantation nor electrical stimulation caused additional damage to IHC synapses.

## 3 Discussion

In this study, intracochlear electrical stimulation, a modality also utilized in clinical CI, demonstrated remarkable ability to counter the reduction in VGLUT1 densities and the accompanying elevation of VGLUT2 densities across all regions of the CN in NIHL guinea pigs. These changes restored VGLUT levels to those observed in normal‐hearing guinea pigs, an effect not achieved with intracochlear implantation alone. These findings suggest that intracochlear electrical stimulation is highly effective in reversing the reduction in auditory innervation and the increase in somatosensory innervation within the CN following NIHL. While we could not directly assess tinnitus due to the lack of validated tinnitus models in permanent severe and profound NIHL, previous studies have linked the reversal of maladaptive plasticity of auditory and somatosensory innervation in the CN to tinnitus relief in noise‐induced tinnitus models.^[^ ^26^, ^27^ ^]^ However, this effect was achieved through distinct methods: one involved bimodal stimulation to alter spike timing‐dependent plasticity by modifying the balance between long‐term potentiation and long‐term depression,^[^ ^27^ ^]^ while the other employed cochlear electrical stimulation to increase auditory inputs by activating the auditory nerve.^[^ ^26^ ^]^ Thus, we hypothesize that CIs, through intracochlear electrical stimulation, may similarly reverse this maladaptive plasticity of auditory and somatosensory innervation, thereby contributing to tinnitus mitigation in patients with severe or profound hearing loss.

Tinnitus can arise from various causes beyond noise exposure, including salicylate toxicity. Salicylate, a nonsteroidal anti‐inflammatory drug, is known to induce tinnitus in some individuals after prolonged aspirin use.^[^ ^28^ ^]^ Administering high doses of sodium salicylate to animal models is a common method to induce transient tinnitus. In contrast to noise‐induced tinnitus, salicylate‐induced tinnitus is reversible. In this study, we used repeated intraperitoneal injections of sodium salicylate (200 mg kg^−1^, twice daily for 14 days) to avoid gastrointestinal side effects from high doses and extend the duration of tinnitus in guinea pigs.^[^ ^29^ ^]^ This extended the tinnitus window for at least 7 days, enabling us to investigate the plasticity of auditory centers and the efficacy of electrical stimulation interventions.

Our findings revealed similarities in VGLUT1 and VGLUT2 density alterations within the CN in both salicylate‐induced tinnitus and NIHL models, despite their differing etiologies. Unlike noise‐induced tinnitus, which arises solely from reduced auditory signaling, salicylate‐induced tinnitus likely involves a central origin as well, given that salicylate crosses the blood‐brain barrier and disrupts central auditory processing. To address this, we bilaterally implanted electrode arrays outside the cochleae of guinea pigs and found that bilateral extracochlear electrical stimulation significantly reduced tinnitus‐like behaviors without affecting hearing thresholds. This suggests that tinnitus relief was not solely due to restoring peripheral auditory function but rather to reversing maladaptive plasticity in the CN via extracochlear electrical stimulation. This aligns with clinical findings showing that many CI patients experience tinnitus relief after prolonged electrical stimulation, even after the device is deactivated.^[^ ^30^, ^31^ ^]^

Inhibitory neural circuits play a crucial role in CN function, integrating auditory and multisensory inputs. Fusiform cells, the primary efferent neurons in the DCN, receive excitatory and inhibitory inputs through complex synapses. Inhibitory neurotransmitters such as glycine and GABA, transported by VGAT, are critical for regulating this activity.^[^ ^24^, ^32^ ^]^ Our study revealed that VGAT densities were reduced in specific CN regions of NIHL and salicylate‐induced tinnitus models compared to normal‐hearing animals. Intracochlear electrical stimulation restored VGAT densities in NIHL animals, and extracochlear stimulation had a similar effect in tinnitus models. These findings suggest that intracochlear and extracochlear electrical stimulation exerted a region‐specific modulation of VGAT expression, with potential implications for reversing inhibitory neural loss and restoring balance in the CN. This restoration was linked to significant reductions in tinnitus behaviors, suggesting that both intracochlear and extracochlear electrical stimulation can rehabilitate disrupted inhibitory circuits in the CN, contributing to tinnitus relief.

Interestingly, the patterns of VGAT density changes varied between NIHL and tinnitus animals across different CN regions. In tinnitus animals, VGAT densities decreased across all five CN regions but were restored after extracochlear stimulation. In contrast, in NIHL animals, significant changes in VGAT densities were not observed in certain CN regions (DCN1 and AVCN), even after intracochlear stimulation. These discrepancies may reflect the distinct underlying mechanisms of NIHL and salicylate‐induced tinnitus onset, as well as the functional differences between CN regions.

While CIs are effective for many patients with hearing loss and tinnitus, not all cases require implantation, as the procedure can cause damage to delicate intracochlear structures. To mitigate these risks, we previously developed a specialized extracochlear electrode array that minimizes harm while providing electrical stimulation along the cochlear axis.^[^ ^26^ ^]^ The use of alternating‐polarity charge‐balanced biphasic electrical pulses and individualized electrical stimulation intensity based on the ECAP threshold can diminish electrochemical toxicity. This study demonstrated that our extracochlear stimulation method had no significant effect on ABR thresholds or inner hair cell synapse numbers, suggesting that it does not damage residual hearing or cochlear structures. Extracochlear implantation elevates high‐frequency hearing thresholds in normal guinea pigs, which may be due to the fact that the implantation disrupts the integrity of the auditory follicle, and the use of glue may alter the vibratory conduction properties of the cochlea. This non‐invasive approach successfully alleviated salicylate‐induced tinnitus in guinea pigs with nearly normal hearing, aligning with our previous findings on noise‐induced tinnitus.^[^ ^26^ ^]^ Combining these findings, the use of multi‐channel extracochlear implants delivering charge‐balanced biphasic electrical pulse stimulation, tailored to the ECAP thresholds, holds immense promise for alleviating tinnitus without compromising the delicate intracochlear structures or existing hearing.

It should be noted that the subjective nature of tinnitus poses a significant challenge, particularly in studies relying on GPIAS, which depends on the animals' hearing capabilities. This issue becomes especially relevant for CI recipients, who typically experience profound hearing loss. As a result, assessing the efficacy of electrical stimulation in these cases necessitates an alternative approach. In this study, we focused on structural changes within the CN of NIHL guinea pigs, correlating these findings with existing data from salicylate‐ or noise‐induced tinnitus models. This dual approach offers a promising avenue for investigating how cochlear implants may mitigate tinnitus, contributing to a more comprehensive understanding of the underlying mechanisms and informing future therapeutic strategies. Due to the lack of practical methods for assessing tinnitus behaviors in guinea pigs with severe hearing loss, further investigation is needed to determine whether the VGLUT changes observed in the CN of NIHL animals are directly associated with tinnitus reduction. Moreover, the development of tinnitus in humans is highly complex, and significant differences exist between animal models and human cases. These differences must be carefully considered when applying these findings to clinical settings.

In summary, this study demonstrates that both intracochlear and extracochlear electrical stimulation can effectively reverse noise‐ or salicylate‐induced disruptions in auditory, somatosensory, and inhibitory innervation in the CN. These therapeutic effects were closely associated with the alleviation of tinnitus‐like behaviors, highlighting the potential of extracochlear electrical stimulation to reverse maladaptive plasticity in the CN and contribute to tinnitus relief while preserving residual hearing.

## 4 Experimental Section

### Experimental Design

Male Dunkin‐Hartley guinea pigs (250–400 g) were used in this study. All animals underwent comprehensive health screenings to confirm they were free of conditions affecting hearing, such as otitis media. The guinea pigs were housed under standard laboratory conditions with unlimited access to food and water, and no exposure to ototoxic drugs was recorded throughout the study. All experimental procedures were approved by the Eye and ENT Hospital of Fudan University ethical committee (No. 2023DW199).

In the **noise‐induced hearing loss (NIHL) experiment**, 15 guinea pigs with confirmed NIHL were randomly divided into three groups:

- **NIHL Control Group (NIHL)**: Five guinea pigs served as controls and received no further intervention.
- **NIHL Intracochlear Implantation Group (NIHL+II)**: Five guinea pigs underwent unilateral intracochlear implantation followed by sham stimulation.
- **NIHL Intracochlear Electrical Stimulation Group (NIHL+IES)**: Five guinea pigs received electrical stimulation following unilateral intracochlear implantation.

Additionally, 15 guinea pigs with normal hearing were included for comparison:

- **Normal Control Group (N)**: Five guinea pigs with normal hearing served as controls.
- **Normal Intracochlear Implantation Group (N+II)**: Five guinea pigs underwent unilateral intracochlear implantation followed by sham stimulation.
- **Normal Intracochlear Electrical Stimulation Group (N+IES)**: Five guinea pigs received electrical stimulation after unilateral cochlear implantation.

In the **salicylate‐induced tinnitus experiment**, 30 guinea pigs were divided into six groups:

- **Saline Control Group (NS)**: Five healthy guinea pigs received daily intraperitoneal saline injections for 14 days.
- **Tinnitus Group (S14)**: Five guinea pigs received twice‐daily intraperitoneal injections of sodium salicylate (200 mg kg^−1^) for 14 days and were euthanized following tinnitus assessment.
- **Recovery Group (R7)**: Five guinea pigs, confirmed to have tinnitus after receiving sodium salicylate for 14 days, were then observed for an additional 7 days without further intervention.
- **Tinnitus Extracochlear Implantation Group (T+EI)**: Five guinea pigs with confirmed tinnitus underwent bilateral extracochlear implantation followed by sham stimulation.
- **Tinnitus Extracochlear Electrical Stimulation Group (T+EES)**: Five guinea pigs with tinnitus received electrical stimulation following bilateral extracochlear implantation.
- **Normal Extracochlear Implantation Group (N+EI)**: Five normal guinea pigs underwent bilateral extracochlear implantation followed by sham stimulation.
- **Normal Extracochlear Electrical Stimulation Group (N+EES)**: Five normal guinea pigs received electrical stimulation following bilateral extracochlear implantation.

### Induction of Noise‐Induced Hearing Loss

To induce permanent high‐frequency hearing loss, guinea pigs were exposed to octave band noise (8–16 kHz) at 106 dB SPL for 8 h, following established protocols.^[^ ^33^, ^34^ ^]^ The noise stimulus was generated digitally using the TDT RPvdsEx system (Tucker‐Davis Technologies) and amplified using a Power Amplifier P9500S. The sound was delivered through four tweeters (TW67, PYRAMID Sound Corporation) positioned 25 cm above the animals. Sound pressure levels were precisely calibrated with a ¼‐inch microphone connected to a sound level meter, ensuring consistent and controlled exposure to the targeted noise frequencies. Hearing loss was assessed 2 weeks post‐exposure using auditory brainstem response (ABR) testing.

### Measurement of Auditory Brainstem Responses

ABR testing was conducted under anesthesia in a shielded chamber. A heating pad was used to maintain the guinea pigs’ body temperature throughout the procedure. Stimuli for ABR measurements included tone bursts at frequencies of 2, 4, 8, 16, 24, and 32 kHz, presented at intensities decreasing from 90 to 20 dB SPL in 5 dB decrements. The stimuli had 2 ms cos^2^ rise/fall times, 1024 repetitions, a 30 Hz presentation rate, and a 3 ms tone pulse duration, generated using SigGenRP and BioSigRP software (Tucker‐Davis Technologies Inc.). Electrode placement for ABR recording was standardized: the reference electrode was positioned at the midpoint of the parietal skull, the recording electrode was placed beneath the mastoid process, and the ground electrode was attached subcutaneously to the back. ABR thresholds were determined by identifying the lowest stimulus level that elicited a wave III response.

### Tinnitus Behavioral Testing

Tinnitus behaviors were assessed using the Gap Prepulse Inhibition of the Acoustic Startle (GPIAS) test, utilizing the RZ6 system (Tucker‐Davis Technologies Inc.) in conjunction with the MED‐ASR‐PRO system (MED‐Associates Company). Guinea pigs were individually placed in a metal cage positioned above a piezoelectric transducer, inside a soundproof box (ENV‐022S, MED) to acclimate them to the experimental environment. Following a 5‐min habituation period, pressure changes caused by the startle reflex were recorded. Acoustic stimuli and background noise were delivered through speakers positioned at the head and tail of the animals, respectively.

The background sound, consisting of 65 dB SPL broadband noise, was continuously played throughout the experiment, except during silent gaps. Four types of background noise were used: narrowband noise with center frequencies of 9, 13, and 17 kHz (2 kHz bandwidth), and wideband noise. Each trial consisted of 20 blocks: 10 blocks where a 50‐µs silent gap was introduced 100 µs before the startle stimulus (20 µs, 95 dB SPL), and 10 blocks without a silent gap. These blocks were presented in random order. Prior to each trial, animals underwent a 5‐min acclimation period, and the inter‐block intervals varied randomly between 30 and 45 s. The GPIAS test utilized four background noise frequencies, with three trials conducted at each frequency as a baseline before sodium salicylate injection and three trials at each frequency 2 weeks post‐injection.

The downward pressure generated by the guinea pigs in response to the startle stimulus was converted into voltage, and the amplitude variation in each block was recorded. To calculate the GPIAS inhibition ratio, the average startle reflex amplitude in silent gap blocks was divided by the average startle reflex amplitude in no‐gap blocks.

Additionally, pre‐pulse inhibition (PPI) tests were conducted simultaneously to account for potential false positives in the GPIAS results due to hearing loss. In the PPI test, the 10 silent gaps were replaced by 75 dB SPL pre‐stimulus sounds, while all other parameters remained the same as in the GPIAS test. The PPI inhibition ratio was calculated by dividing the average startle reflex amplitude in the pre‐stimulus blocks by the average amplitude in the no‐gap blocks. The tinnitus index (TI) was determined by subtracting the GPIAS inhibition ratio from the PPI inhibition ratio.

### Intracochlear and Extracochlear Implantation of Electrode Array

The implant used for both intracochlear and extracochlear electrical stimulation (ES) consisted of an active electrode array with four ring electrodes, a reference electrode (MP1), and a grounding electrode (MP2), as described in a previous study.^[^ ^26^ ^]^ Each electrode measured 0.45 mm in diameter and 0.30 mm in length, with 0.75 mm spacing between them. The external terminal of the cochlear implant (CI) was connected to a pulse generator via a flexible cable and USB port. Guinea pigs were anesthetized with tiletamine hydrochloride (16 mg kg^−1^) and zolazepam hydrochloride (16 mg kg^−1^) and placed on a heating pad, with additional anesthesia administered as needed.

For intracochlear implantation, a postauricular incision was made to expose the auditory bulla. A small bony window was carefully created on the bulla wall to gain access to the round window membrane. Four electrodes were then inserted into the scala tympani via an incision in the round window (see Figure 1B). A blood‐free muscle fragment was placed over the round window to protect the cochlear structure, while MP1 and MP2 electrodes were implanted subcutaneously. All intracochlear implants were unilateral (see Figure 5A).

For extracochlear implantation, electrodes were implanted bilaterally according to a previously established protocol.^[^ ^26^ ^]^ The bulla was opened to expose the cochlear wall, where the electrode arrays were positioned and secured using medical glue. Electrode impedance was measured to confirm proper contact with the cochlear wall and correct placement of MP1 and MP2 on each side. Post‐surgery, electrode impedance and electrically evoked compound action potentials (ECAPs) were promptly tested to ensure correct electrode placement and functionality.

### ECAP Measurement and Electrical Stimulation

Prior to electrical stimulation, ECAP (electrically evoked compound action potential) threshold levels were individually determined for each stimulating electrode using a sound processor and MAP V3.00 software (Shanghai Listent Medical Technology Co., Ltd). This system, integrated into the cochlear implant (CI), included an analog amplifier and digital converter, providing a 40 dB signal amplification (100‐fold increase). The ECAP recording stimulus consisted of charge‐balanced biphasic pulses with a 32‐µs phase duration and a 9‐µs interphase gap, delivered at 40 pulses per second (pps) for 50 iterations. Consistent parameters were maintained throughout, including a default gain of 40 dB, a 50‐µs delay, and a 20 kHz sampling frequency. To minimize stimulation artifacts, electrical stimulation was alternated in leading‐phase polarity.

The current levels increased in 5‐CL (current level) steps, ranging from 110 to 180 CL (µA = 18 * 100^[CL/255]), to determine ECAP thresholds. Waveforms for all stimulus steps were recorded using MAP software for analysis. ECAP thresholds were defined as waveforms with amplitudes of ≥50 µV.

Electrical stimulation was delivered using charge‐balanced biphasic cathodic‐leading pulses (32 µs/phase, 9 µs interphase gaps) at a frequency of 12 kHz across the four electrodes, utilizing a custom‐built charge generator. Stimulation was provided in monopolar mode at intensities corresponding to the ECAP thresholds. The electrical stimulation regimen consisted of 2‐h sessions per day over a period of 3 days.

### Immunohistology

Guinea pigs were anesthetized and subjected to transcardial perfusion with 500 mL of 0.1 m PBS followed by 500 mL of 4% paraformaldehyde (PFA, pH 7.2–7.4). The cochlea was carefully removed and immersed in 4% PFA for 1 h, while the brainstem was excised and immersed in 4% PFA overnight at 4 °C. After fixation, the brainstem was gradually dehydrated over the course of 1 week and embedded in Tissue‐Tek (Sakura Finetek USA, Inc.).

A small hole was made on the right side of the brainstem to distinguish lateral differences. Coronal brainstem sections (25 µm thick) were obtained using a cryostat (Leica CM 3050S). Four series of these sections were collected and treated with a blocking solution (PBS containing 1% normal goat serum and 0.1% Triton‐X) for 30 min. The sections were then incubated overnight at 4 °C with primary antibodies against VGLUT1 (Synaptic Systems, 1:1000), VGLUT2 (Synaptic Systems, 1:1000), and VGAT (Synaptic Systems, 1:500), diluted in the blocking solution. The next day, the sections were incubated at room temperature for 2 h with fluorescent secondary antibodies (Alexa Fluor Plus 555 and Alexa Fluor 488, both from Thermo Fisher Scientific; 1:500 in blocking solution).

For cochlear analysis, the sections of basilar membrane were treated with a blocking solution (PBS with 10% normal goat serum and 0.2% Triton‐X) at 37 °C for 1 h. They were then incubated overnight at 4 °C with primary antibodies against CtBP2 (BD Biosciences, 1:400), GluR2 (Millipore, 1:1000), and Myosin 7a (Proteus Biosciences, 1:800). After primary antibody incubation, the sections were treated with fluorescent secondary antibodies: Alexa Fluor 488‐conjugated goat anti‐mouse (IgG1, 1:1000, Thermo Fisher Scientific), Alexa Fluor 555‐conjugated goat anti‐mouse (IgG2a, 1:2000, Thermo Fisher Scientific), and Alexa Fluor 647‐conjugated donkey anti‐rabbit (1:800, Jackson ImmunoResearch) at room temperature for 1.5 h.

All sections were mounted onto microscope slides and covered with Fluoromount‐G (Electron Microscopy Sciences) and a coverslip. Negative controls, in which the primary antibody was omitted, were included to confirm the specificity of the staining.

### Fluorescence Image Acquisition and Processing

Immunofluorescence imaging of the cochlear nucleus was performed using a laser scanning confocal microscope (TCS SP8, Leica) equipped with 63x and 40x oil‐immersion objectives and a 3.00x digital zoom. Fluorescence was stimulated at wavelengths of 488, 561, and 633 nm. Z‐stack projections of the cochlear nucleus were captured at 3.0‐µm intervals. For each subregion in cochlear nucleus, at least three different micrographs were scanned from caudal to rostral sections. Images were captured at 40x magnification, except for those of the DCN1 stained with VGLUT1, which were taken at 63x magnification.

Quantification was performed as previously described.^[^ ^19^, ^35^ ^]^ Image analysis was conducted using ImageJ software (version 1.53t, NIH, USA). RGB images were converted to 8‐bit grayscale, and automated thresholding was applied to segment puncta: the “Intermodes Auto Threshold” was used for 40x images, while the “Bernsen Auto Local Threshold” was used for 63x images. The “Watershed” function was then applied to separate fused puncta. Puncta counts were calculated using the “Analyze Particles” function in ImageJ and normalized to image area, yielding puncta density (see Figure 2B). This objective image analysis protocol was validated by two independent observers to ensure that the automated counts accurately represented manual counts for all regions of the cochlear nucleus.

For the basilar membrane, images were taken at 1.0‐µm intervals and subjected to z‐axis reconstruction using ImageJ. Synapse counting was performed by selecting five adjacent inner hair cells (IHCs) per micrograph and determining the average puncta count per cell. Consistent microscope settings were maintained throughout the experiment, and the researchers performing puncta counts were blinded to the experimental groupings.

### Statistical Analysis

Data were analyzed and presented using GraphPad Prism software (version 8.0, RRID: SCR_0 02798). Depending on the dataset, one‐way ANOVAs, Brown‐Forsythe and Welch ANOVA tests, Welch's t‐test, Tukey‐Kramer post hoc test, and Student's t‐test were applied as appropriate. Detailed descriptions of the specific statistical tests used for each analysis are provided in the figure legends and the Results section. Statistical significance was set at P < 0.05.

## Conflict of Interest

The authors declare no conflict of interest.

## Author Contributions

M.C., S.F., and J.M. contributed equally to this work. S.L. conceptualized and supervised the project. M.C., S.F., J.M.B, C.Z., and S.L. contributed to methodology. M.C., S.F., J.M., L.H., N.T., and W.L. contributed to Investigation. M.C., S.F., and S.L. visualized the data. M.C., S.F., and J.M. wrote the original draft. S.L. and M.C. responded to review and editing.

## Data Availability

All data needed to evaluate the conclusions in the paper are present in the paper.
